# Epigenetics of oral and oropharyngeal cancers (Review)

**DOI:** 10.3892/br.2020.1290

**Published:** 2020-03-06

**Authors:** Daniela Russo, Francesco Merolla, Silvia Varricchio, Giovanni Salzano, Giovanni Zarrilli, Massimo Mascolo, Viviana Strazzullo, Rosa Maria Di Crescenzo, Angela Celetti, Gennaro Ilardi

Biomed Rep 9:275-283, 2018; DOI: 10.3892/br.2018.1136

Following the publication of the above review, the authors have noted that acronym ‘SS‘ should not have been featured in the Authors' contributions section of the Declarations; this was an error made during the transcription of the article, and there is no such author with these initials on the Review. The authors apologize to the readership for any confusion this may have caused.

